# Blood culture time to positivity in pediatric patients with bloodstream infection in rural Gambia

**DOI:** 10.1016/j.ijregi.2025.100606

**Published:** 2025-02-18

**Authors:** Isaac Osei, Baleng Mahama Wutor, Alieu Kuyateh, Ousman Barjo, Golam Sarwar, Mayowa Omotosho, Williams Oluwatosin Adefila, Yusuf Abdulsalam Olawale, Keita Modou Lamin, Ilias Hossain, Babila G. Lobga, Muhammed Wally, Morr Cham, Minteh Molfa, Rasheed Salaudeen, Grant A. Mackenzie

**Affiliations:** 1 Medical Research Council Unit The Gambia at London School of Hygiene & Tropical Medicine, Banjul, The Gambia; 2 Department of Disease Control, Faculty of Infectious and Tropical Diseases, London School of Hygiene & Tropical Medicine, London, UK; 3 Murdoch Children's Research Institute, Melbourne, Australia; 4 Department of Paediatrics, University of Melbourne, Melbourne, Australia

**Keywords:** Bloodstream infections, Children, The Gambia, Blood cultures, Time-to-positivity

## Abstract

**Objectives:**

There is a lack of data on the time to blood culture positivity (TTP) in pediatric populations in low-income countries. We aimed to assess the host and pathogen factors associated with TTP in children aged under 5 years in rural Gambia.

**Methods:**

Between September 2019 and December 2023, we collected blood cultures from children under 5 years with suspected bloodstream infections. We determined the TTP from the time of culture incubation to when bacterial growth was first detected.

**Results:**

Overall, 547 invasive bacteria pathogens were evaluated. The median TTP was 19.2 hours and 70%, 76%, 89%, and 96% of cases had TTP at 24, 36, 48, and 72 hours, respectively. *Streptococcus pneumoniae* had the shortest median TTP (17.4 hours), whereas *Neisseria* species had the longest (45 hours). TTP was dependent on the pathogen and independent of age, sex, temperature, clinical outcome, nutritional status, and length of hospital stay. Gram-positive bacteria had shorter TTP than gram-negative bacteria (18.6 vs 19.6 hours, *P* < 0.01).

**Conclusions:**

In rural Gambia, most blood cultures from pediatric patients would turn positive within 48 hours of incubation. A maximum of 48 hours of observation after the commencement of antibiotic therapy in hospitalized children may be sufficient for clinicians to receive feedback on blood culture results.

## Highlights

• The median time to positivity was 19.2 hours.
• At 24, 36, 48, and 72 hours, 70%, 76%, 89%, and 96% of cases, respectively, had time to positivity (TTP).
• TTP was dependent on the pathogen and independent of host factors.
• Gram-positive bacteria had shorter TTP than gram-negative bacteria.
• TTP was not a predictor of poor clinical outcomes.

## Introduction

Bloodstream infections (BSIs) are a major cause of morbidity and mortality among all age groups worldwide. The term BSI generally refers to the growth of a microorganism from a blood culture obtained from a patient with clinical signs of infection and where contamination has been ruled out [1]. In 2015, 31.5 million people were estimated to be affected by BSI, with an attributable 5.3 million deaths worldwide [2]. BSI may be associated with pneumonia, sepsis, and meningitis. Bacteria such as *Streptococcus pneumoniae, Escherichia coli, Staphylococcus aureus, Klebsiella pneumoniae*, and *Pseudomonas aeruginosa* are some of the common pathogens implicated in BSIs [3].

Blood cultures are routinely performed in patients suspected of BSI to identify the causative organism and inform appropriate antibiotic therapy. Delays in antibiotic treatment for patients with BSIs may lead to bad outcomes [[4], [5], [6], [7]]. In addition, inappropriate use of antibiotics is a major cause of antibiotic resistance, which is currently a major public health concern [8,9]. Early detection of positive blood culture is critical for patient management and may impact clinical outcomes [[10], [11], [12]].

The time to positivity (TTP) of blood culture, defined as the time from the start of culture incubation to the detection of bacterial growth by an automated system, has been identified as an important indicator of patient outcomes [13]. A recent systematic review and meta-analysis of the impact of TTP on patient outcomes showed that a short TTP is an important predictor of mortality and septic shock in BSIs [14]. A shorter TTP may indicate a higher bacteria load in the blood, which may suggest a severe infection [15,16].

While there has been an increase in the number of studies on TTP in the last decade, most of these studies have been conducted in high-income countries [14]. Although sub-Saharan Africa (SSA) has the highest burden of BSIs [3], a recent systematic review on TTP demonstrated the lack of African data. There was no study from SSA among the 24 eligible studies included in the systematic review [14]. In addition, there is a dearth of TTP data on pediatric age groups. The lack of data in this age group has been identified as a limitation to the use of available data on TTP for optimal use of antibiotic treatment for pediatric patients suspected of BSIs [17]. To address these gaps, we aim to assess host and pathogen factors associated with TTP in children under 5 years with positive blood cultures admitted to health facilities in rural Gambia with suspected BSIs.

## Materials and Methods

### Study design and population

This retrospective cohort study was performed at 11 health facilities in the Gambia's Central and Upper River regions. We analyzed the TTP in children under 5 years enrolled in the Pneumococcal Vaccine Schedules (PVS) trial with invasive bacteria pathogenic blood cultures between September 9, 2019 and December 31, 2023. The Gambia is a small West African country with a population of about 2.7 million. The Gambia is classified as a low-income country with a gross domestic product per capita of US $808 [18]. The public health system operates with basic human and physical resources. There are two main hospitals within the study area and several health centers and private pharmacies. Antibiotics can be bought over the counter without a prescription [19].

The PVS trial compared a reduced dose schedule of the pneumococcal conjugate vaccine with the standard dose in infants. The protocol of the trial has been described previously [20]. According to standardized definitions, children under 5 years who presented to health facilities within the study area were screened for syndromes suggestive of pneumonia, sepsis, meningitis, and other medical conditions. Those with any of these diagnoses and who were admitted had a blood culture collected, cerebrospinal fluid if they had suspected meningitis, and lung aspirate if they had pneumonia with a large peripheral consolidation. In most cases, samples were collected before the administration of antibiotics. In the few instances where antibiotics were initiated before sample collection, the procedure was recorded to reflect the same. The date and time of sample collection were recorded in the electronic medical record system (EMRS) and the clinical logbook.

Patients with polymicrobial growth with repeat growth that had missing clinical or laboratory data were excluded.

### Clinical and laboratory procedures

Blood culture was taken from eligible children by study nurses and clinicians. After washing their hands and wearing gloves, the study staff applied a tourniquet and thoroughly cleaned the venipuncture area. The cleaning was done using sterile cotton soaked in 70% ethyl alcohol in circular motions from the center to the periphery. After allowing the alcohol to dry, 1-5 ml of blood was taken with a 10 ml syringe. The used needle was replaced with a new one, and the blood sample was inoculated into a blood culture bottle. Quality control was routinely conducted during sample collection to ensure consistency and reduce contamination.

Two types of blood culture bottles were used: BD BACTEC Peds Plus/F Culture Vials (Becton, Dickinson and Company, USA) and BACT/ALERT PF Plus (BioMérieux, USA). In most cases, the blood culture bottles were transported to the laboratory within two hours of collection. Blood samples collected in the evenings were stored at room temperature at the health facilities and transported to the laboratory early the next morning. The laboratory logged samples that were brought in and immediately placed the blood culture bottles in automated BD BACTEC (Becton Dickinson, Maryland, USA) or BACT/ALERT (BioMérieux, USA) machines for a maximum period of five days. Positive samples with signals were removed and sub-cultured on blood agar, chocolate agar, or MacConkey agar. Bacteria were identified using standard microbiological procedures and biochemical tests (API, BioMérieux, USA). The date and time of culture incubation and the date and time when a positive signal was detected were recorded. In addition to the blood culture samples, whole blood samples were also collected from eligible patients seen at the two hospitals. Plasma aliquots from the whole blood were tested for antibiotic activity using a fully sensitive bacterial control strain. Consistent with the definition broadly used in TTP studies, we defined TTP as the time from incubation to the time when a positive signal is detected [17,21]. Positive cultures that grew *Bacillus* spp., *Corynebacterium* spp., *Micrococcus* spp*.,* coagulase-negative *Staphylococcus, viridans* group *Streptococci,* and *Propionibacterium acnes* were considered as contaminants [22].

### Statistical analysis

We extracted the patient's clinical data from the EMRS and the laboratory data from the laboratory EMRS. All extracted data were stored in Microsoft Excel and assessed for missing data and errors. The data were cleaned and exported to STATA 18 (StataCorp, College Station, TX, USA). The TTP was calculated as the difference between the time the sample was placed in the automated blood culture monitoring machines and the time a positive signal was detected. We performed descriptive analyses of the distribution of sociodemographic, clinical, and pathogen factors using frequencies and proportions for categorical and binary variables and means and medians (interquartile range [IQR]) for continuous variables with 95% confidence intervals (CIs) and *P-values* of likelihood ratio testing. We determined the proportion of TTP at thresholds of ≤ 24 hours, > 24 to ≤ 36 hours, > 36 to ≤ 48 hours, and > 48 to ≤ 72 hours. We fitted a generalized linear regression model comparing the median TTP between pathogens. We performed a multivariable linear regression analysis to examine the association between age, sex, temperature at presentation, clinical outcome, nutritional status, and length of hospital stay on the mean TTP of the four most common pathogens. A sensitivity analysis excluding samples collected on dates different from the date of blood culture bottle incubation was performed to determine whether the duration between sample collection and incubation influenced the TTP. All analyses were performed using Stata version 18.0.

### Ethical approval

The PVS clinical trial was approved by the Gambia Government/Medical Research Council Unit The Gambia Joint Ethics Committee (reference: 1577), and the London School of Hygiene and Tropical Medicine Ethics Committee (reference: 14515). Written informed consent to participate was obtained from all enrolled participants.

## Results

### Characteristics of the sample

**Figure 1:**
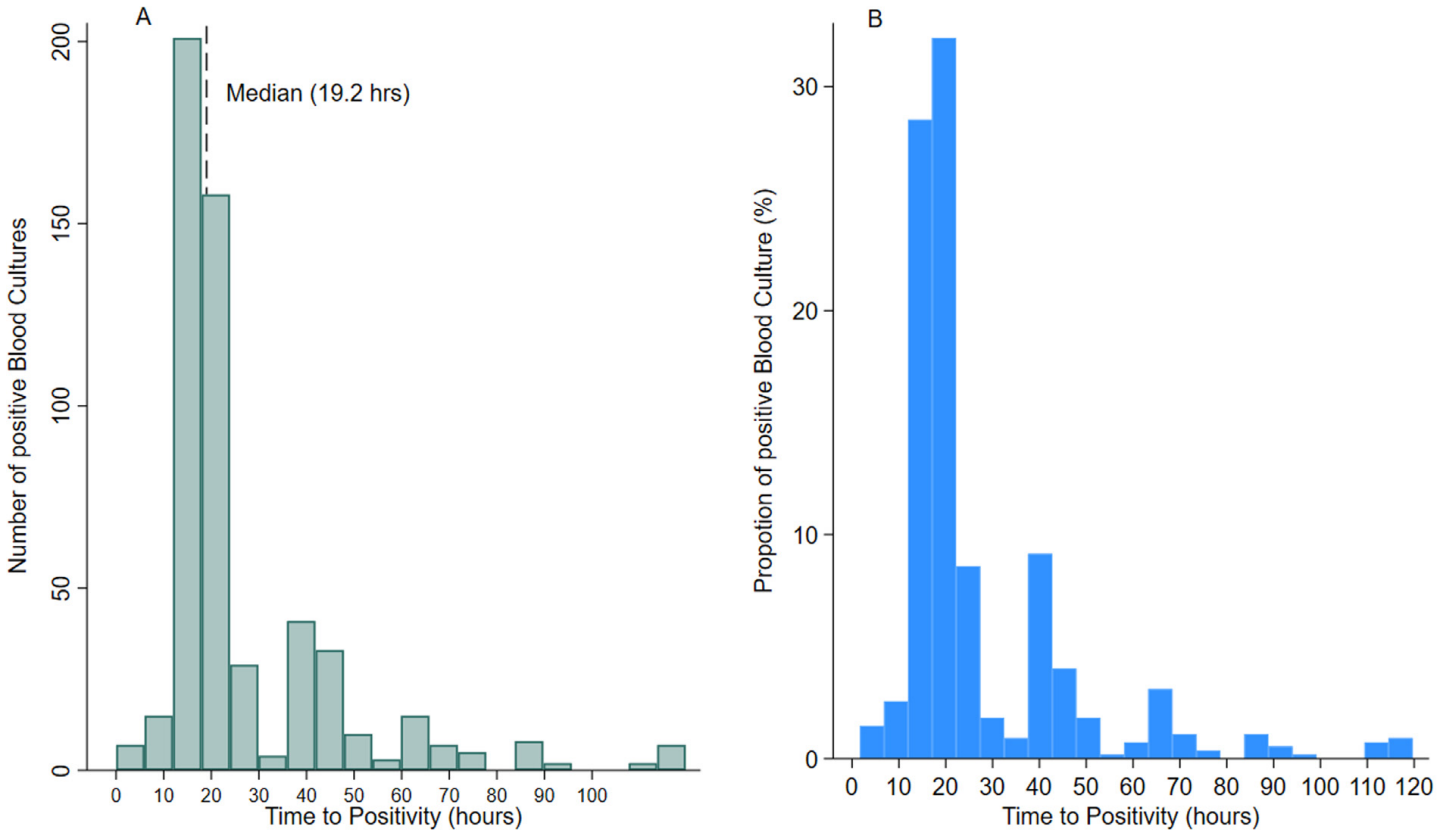
The distribution and proportions of positive blood cultures and TTP. (a) The distribution of the overall number of positive blood cultures and TTP (with median TTP shown as a dashed line). (b) The proportion of positive blood culture with TTP. TTP, time to positivity.

From September 2019 to December 2023, 717 patients with invasive pathogenic blood cultures were analyzed. Among these, 36 patients had polymicrobial blood culture growth, 24 had repeat growth, 57 had missing clinical data, and 53 had missing blood culture TTP records due to mechanical factors and were excluded. After exclusion, 547 invasive bacteria pathogenic cultures remained for analysis. The median age at blood draw was 12 months (IQR, 4-21). There were slightly more males (55%) than females. About a third (35%) of the samples were collected from neonates and 40% had temperatures ≥ 38.0°C. Most (88%) of the patients recovered fully, were not malnourished (71%) and were admitted to the hospital for less than 7 days (82%). About a fifth (22%) self-reported antibiotic use within a week before presentation at the health facility, most of the samples (76%) were collected at the hospital, and 53% of the inoculated blood sample volumes were > 1.5 mls. Antibiotic activity detection testing was performed on samples collected from the two hospitals. Out of 418 available results, only 13% were positive for antibiotic activity, with 61 results in total. The median TTP was 19.2 hours (IQR 16.4 - 30.6). There were more gram-negative isolates (59.6%) than gram-positives. The median TTP was shorter for gram-positive bacteria than for gram-negative bacteria (18.6 vs 19.6, *P* <0.01). The median TTP was similar across all sociodemographic and clinical indicators (Table 1 and Figure 1). The sensitivity analysis that excluded samples collected on different dates relative to the date of incubation showed similar results (Supplementary Table 1).

**Table 1:** Sociodemographic, clinical characteristics, and TTP.

Characteristic	N^a^ (%)	TTP, median (IQR), hours		*P*-value (likelihood ratio test)
Total no.	547 (100)	19.2 (16.4-30.6)		
*Age at blood draw, months*				
Neonate	192 (35.1)	19.9 (16.7-37.3)		
1-11 months	154 (28.2)	19.0 (16.6-37.6)		0.37
12-23 months	127 (23.2)	19.2 (16.1-34.6)		
24-59 months	74 (13.5)	18.4 (15.7-27.8)		
*Sex*				
Male	298 (54.5)	19.1 (16.3-27.2)		
Female	249 (45.5)	19.4 (16.6-35.9)		0.94
*Temperature at blood draw,°C*				
<38.0	325 (59.4)	19.2 (16.4-28.0)		
≥38.0	222 (40.6)	19.2 (16.5-37.6)		0.89
*Clinical outcome*				
Alive	482 (88.1)	19.2 (16.4-29.2)		
Dead	65 (11.9)	19.1 (16.3-39.2)		0.79
*Severe malnutrition, (Z score <−3 SD)*				
Yes	147 (29.2)	19.6 (16.7-28.9)		
No	356 (70.8)	19.0 (16.3-28.8)		0.61
*Length of hospital stay, days*				
<3 days	207 (38.8)	19.0 (16.7-33.5)		
3-6 days	229 (43.0)	19.2 (16.2-26.4)		0.51
≥7 days	97 (18.2)	19.5 (16.2-39.9)		
*Self-reported antibiotic use within past 1 week*				
No	420 (78.1)	19.9 (16.4-29.2)		
Yes	118 (21.9)	19.1 (16.5-34.0)		0.50
b*Antibiotic activity detected in sample*				
Absent	53 (86.9)	16.2 (15.8-19.1)		
Present	8 (13.1)	19.5 (15.9-23.5)		0.10
*Type of health facility*				
Health center	129 (23.6)	19.3 (16.6-38.8)		
Hospital	418 (76.4)	19.2 (16.3-28.3)		0.35
*Volume of blood sample, ml*				
≤ 1.5	231 (47.4)	18.4 (16.3-27.8)		
> 1.5	256 (52.6)	19.3 (16.6-27.3)		0.34
*Pathogen category*				
Gram-positive	221 (40.4)	18.6 (16.0-24.0)		
Gram-negative	326 (59.6)	19.6 (16.6-39.6)		<0.01

TTP, time to positivity.

a Missing values: severe malnutrition = 44; length of hospital stay = 14; antibiotic use = 9; antibiotic activity detection test= 357; volume of blood sample = 60.

b Assessed only in children who presented to the hospital.

### TTP thresholds of blood cultures (TTP ≤24 hours, >24 to ≤36 hours, >36 to ≤48 hours, and >48 to ≤72 hours)

At 24 and 48 hours, most of the blood cultures, 70% and 89%, had turned positive, respectively. Just about 11% of blood cultures signaled positivity later than 48 hours of incubation. There was no difference in the proportion of blood cultures which turned positive within 24 hours by age, sex, and temperature at blood draw. However, proportions with TTP ≤ 24 hours were higher in those with good clinical outcomes (70%), those who had shorter hospital stays (71%), those with antibiotic activity present (87%), and gram-positive pathogens (75%). At 48 hours, ≥ 90% of blood cultures from females, neonates, and older children had turned positive (Table 2).

**Table 2:** Patient characteristics with bloodstream infection episodes by TTP thresholds.

Variable	TTP threshold (hours) (%)				
	≤24 hours	>24 to ≤36	>36 to ≤48 hours	>48 to ≤72 hours	
Overall	69.8%	75.9%	89.2%	95.8%	
*Age at blood draw, months*					
Neonate	67.7%	75.0%	92.2%	96.9%	
1-11 months	71.4%	74.7%	87.7%	95.4%	
12-23 months	69.3%	75.6%	84.2%	93.7%	
24-59 months	73.0%	81.1%	93.2%	97.3%	
*Sex*					
Male	70.5%	76.5%	87.6%	95.3%	
Female	69.1%	75.1%	91.2%	96.4%	
*Temperature at blood draw,°C*					
<38.0	70.5%	76.6%	88.6%	96.7%	
≥38.0	68.9%	74.8%	90.1%	96.0%	
*Clinical outcome*					
Alive	70.1%	76.3%	89.6%	96.1%	
Died	67.7%	72.3%	86.1%	93.9%	
*Severe malnutrition, (Z score <−3 SD)*					
Yes	68.0%	77.6%	87.1%	95.9%	
No	71.6%	75.8%	89.0%	95.2%	
Length of hospital stay, days					
<3 days	70.5%	75.9%	87.4%	94.2%	
3-6 days	71.6%	78.6%	90.8%	97.8%	
≥7 days	65.0%	71.1%	90.7%	94.9%	
*Self-reported antibiotic use within past 1 week*					
No	69.3%	75.2%	88.8%	96.4%	
Yes	71.2%	77.1%	89.8%	93.2%	
*Antibiotic activity detected in sample*					
Absent	75.5%	79.2%	92.4%	98.1%	
Present	87.5%	NA	100%	NA	
*Type of health facility*					
Health center	66.7%	74.4%	84.5%	95.4%	
Hospital	70.8%	76.3%	90.7%	95.9%	
*Volume of blood sample, ml*					
≤1.5	70.6%	77.1%	89.6%	96.5%	
>1.5	71.1%	77.3%	89.1%	96.1%	
*Pathogen category*					
*Gram-positive*	75.1%	81.9%	92.3%	97.3%	
Gram-negative	66.3%	71.8%	87.1%	94.8%	

NA, no observation; TTP, time to positivity.

### Distribution of pathogens and TTP

**Figure 2:**
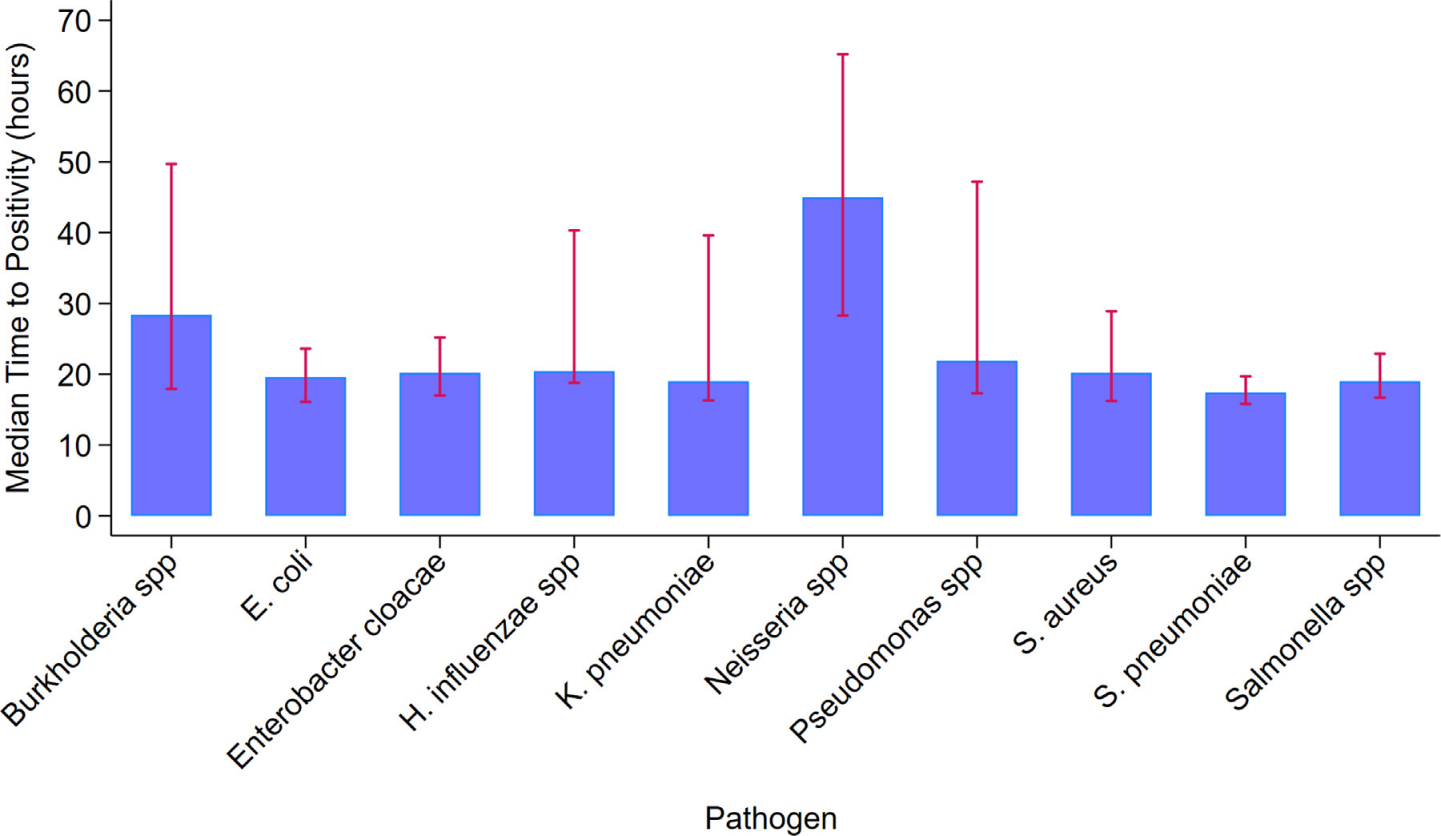
Top 10 pathogens and median time to positivity. Bars show the estimated median time to positivity in hours. Error bars show interquartile ranges (interquartile range).

The top five most prevalent pathogens isolated in blood culture were *Staphylococcus aureus* (26.9%), *K. pneumoniae* (18.8%), *S. pneumoniae* (11.9%), *E. coli* (8.0%), and Salmonella species. Compared with the most prevalent pathogen (*S. aureus*), *S. pneumoniae* had a shorter TTP (median 17.4, IQR: 15.8 - 19.7, *P* <0.01), whereas Burkholderia species (median 28.4, IQR: 17.9 - 49.7, *P* <0.01) had longer TTP. Neisseria species (median 45.0, IQR: 28.3 - 65.2) had the longest TTP (Table 3 and Figure 2).

**Table 3:** Pathogen distribution and median TTP between pathogens, obtained from a generalized linear regression model.

Pathogen	No. (%) N = 547	Time to positivity, median (interquartile range), hours	*P*-value
*Staphylococcus aureus*	147 (26.9)	20.2 (16.2-28.9)	1 [Reference]
*Klebsiella pneumoniae*	103 (18.8)	19.0 (16.3-39.6)	0.55
*S. pneumoniae*	65 (11.9)	17.4 (15.8-19.7)	**<0.01**
*Escherichia coli*	44 (8.0)	19.6 (16.1-23.6)	0.59
*Salmonella spp*	37 (6.8)	19.0 (16.7-22.9)	0.09
*Burkholderia spp*	29 (5.3)	28.4 (17.9-49.7)	**<0.01**
Coliform *spp*^a^	28 (5.1)	19.1 (16.8-39.3)	0.71
*Enterobacter cloacae*	20 (3.7)	20.2 (17.0-25.2)	0.29
*Raoultella spp*	16 (2.9)	20.4 (16.4-26.4)	0.71
*Pseudomonas spp*	16 (2.9)	21.9 (17.3-47.2)	0.49
*Serratia marcescens*	8 (1.5)	22.3 (18.1-31.5)	0.62
*Moraxella spp*	7 (1.3)	25.7 (19.4-68.6)	0.17
*Haemophilus influenzae spp*	6 (1.1)	20.4 (18.8-40.3)	0.77
*Neisseria spp*	6 (1.1)	45.0 (28.3-65.2)	0.07
*Streptococcus pyogenes*	5 (0.9)	17.8 (15.4-17.9)	0.19
*Streptococcus gr B (agalactiae)*	4 (0.7)	21.9 (16.9-25.6)	0.66
*Pantoea spp*	4 (0.7)	17.5 (16.5-18.3)	0.33
*Acinetobacter spp*	2 (0.4)	40.9 (18.4-63.4)	0.40

TTP, Time to positivity.

a Morphologically identified species but final species identification was not performed. Model adjusted for age at blood draw, sex, history of antibiotic use in the past week, and sample volume.

### Factors associated with mean TTP of most prevalent pathogens

For patients with *S. aureus* bacteremia, the TTP of blood cultures for those aged 24-59 months was shorter than the younger age group. The blood cultures of those aged 1-11 months had TTP that was 1.6 times (Geometric Mean Ratio (GMR) 1.6 [95% CI: 1.2 - 2.2]) longer than those aged 24-59 months. Blood cultures from children who died of *S. aureus* infection took longer to grow (GMR 1.5, [95% CI: 1.1 - 2.2]) than those who fully recovered. The TTP of blood cultures from children aged 12-23 months with *K. pneumoniae* infection was shorter (0.4, [95% CI: 0.1 - 0.9]) than cultures from those aged 24-59 months. Children with *S. pneumoniae* bacteremia who presented with temperature ≥ 38.0°C had longer TTP than those who presented with temperatures of < 38.0°C. There were no significant associations found between sex, length of hospital stay, malnutrition status, and TTP of the four most prevalent pathogens (Table 4).

**Table 4:** Relative geometric mean TTP of top four pathogens, obtained from a linear regression model.

Characteristic	*Staphylococcus aureus*		*Klebsiella pneumoniae*		*Streptococcus pneumoniae*		*Escherichia coli*	
	GM TTP 95% CI	GM ratio 95% CI	GM TTP 95% CI	GM ratio 95% CI	GM TTP 95% CI	GM ratio 95% CI	GM TTP 95% CI	GM ratio 95% CI
*Age at blood draw, months*								
Neonate	22.0 (18.7-25.9)	**1.4 (1.0-1.9)**	23.9 (21. 2-27.0)	0.5 (0.2-1.3)	17.3	1 (ref)	24.0 (17.4-33.1)	1.2 (0.7-1.8)
1-11 months	25.3 (21.4-29.9)	**1.6 (1.2-2.2)**	27.9 (18.9-41.2)	0.6 (0.3-1.6)	16.7 (14.2-19.5)	1.0 (0.3-3.1)	18.1 (14.1-23.1)	0.9 (0.5-1.4)
12-23 months	23.7 (19.2-29.3)	**1.5 (1.1-2.1)**	15.4 (9.5-24.9)	**0.4 (0.1-0.9)**	19.1 (14.1-25.9)	1.1 (0.3-3.6)	27.4 (18.1-41.4)	1.3 (0.8-2.1)
24-59 months	15.9 (12.2-21.0)	1 (ref)	43.6 (6.2 - 306. 5)	1 (ref)	14.6 (10.9 - 19.7)	0.8 (0.2 – 2.8)	20.7 (16.9 - 25.3)	1 (ref)
*Sex*								
Male	21.7 (18.7-25.1)	1 (ref)	25.5 (21.6 - 30.1)	1 (ref)	19.1 (16.0 - 22.8)	1 (ref)	21.8 (16.9 - 28.1)	1 (ref)
Female	23.5 (20.7-26.6)	1.1 (0.9-1.3)	20.8 (17.4 - 24.9)	0.8 (0.6 - 1.0)	15.1 (12.1 - 18.7)	0.8 (0.6 - 1.0)	23.5 (19.1 - 28.9)	1.1 (0.8 - 1.5)
*Temperature at blood draw, °C*								
<38.0	22.7 (20.1-25.7)	1 (ref)	24.6 (21.5 - 28.2)	1 (ref)	13.2 (10.1 - 17.2)	1 (ref)	21.2 (17.8 - 25.3)	1 (ref)
≥38.0	22.2 (18.9-25.9)	0.9 (0.8 -1.2)	20.3 (15.5 - 26.6)	0.8 (0.6 - 1.1)	20.2 (17.7 - 23.0)	**1.5 (1.2 - 2.0)**	24.9 (18.4 - 33.8)	1.2 (0.8 - 1.6)
*Clinical outcome*								
Alive	21.9 (19.8-24.1)	1 (ref)	23.7 (20.5 - 27.4)	1 (ref)	16.7 (14.3 - 19.5)	1 (ref)	22.4 (18.6 - 26.9)	1 (ref)
Died	32.9 (21.9-49.5)	1.5 **(**1.1-2.2)	22.7 (18.1 - 28.5)	0.9 (0.7 - 1.3)	19.4 (14.5 - 25.9)	1.2 (0.8 - 1.8)	24.0 (16.8 - 34.4)	1.1 (0.7 - 1.7)
*Severe malnutrition, (Z score <−3 SD)*								
Yes	23.7 (18.3-30.7)	1 (ref)	23.8 (19.5 - 29.1)	1 (ref)	15.2 (11.8 - 19.5)	1 (ref)	26.2 (19.3 - 35.6)	1 (ref)
No	21.7 (19.5-24.3)	0.9 (0.7-1.2)	23.7 (19.6 - 28.7)	1.0 (0.7 - 1.3)	17.9 (15.1 - 21.2)	1.2 (0.8 - 1.6)	21.3 (17.5 - 26.1)	0.8 (0.6 - 1.2)
*Length of hospital stay, days*								
<3 days	21.8 (18.6-25.6)	1 (ref)	22.4 (17.9 - 28.0)	1 (ref)	17.8 (14.6 - 21.6)	1 (ref)	27.4 (20.9 - 36.0)	1 (ref)
3-6 days	22.3 (18.9-26.2)	1.0 (0.8-1.3)	22.0 (18.9 - 25.8)	0.9 (0.7 - 1.3)	17.9 (14.5 -22.3)	1.0 (0.8 - 1.3)	18.9 (15.6 - 23.1)	0.7 (0.5 - 1.0)
≥7 days	22.7 (18.8-27.4)	1.0 (0.8-1.4)	28.8 (22.4 - 36.9)	1.3 (0.9 - 1.8)	16.3 (14.8 - 18.0)	0.9 (0.6 - 1.4)	17.5 (14.7 - 20.7)	0.6 (0.4 - 1.0)

CI = confidence interval; GM = geometric mean; TTP = time to positivity.

## Discussion

In this study, we assessed host and pathogen factors associated with TTP in children under 5 years with positive blood cultures admitted to health facilities in rural Gambia with suspected sepsis, pneumonia, meningitis, and other medical problems. Approximately nine out of ten blood cultures turned positive within 48 hours of incubation. The median TTP was 19.2 hours (IQR 16.4 - 30.6). TTP was dependent on the pathogen and was independent of age, sex, temperature at presentation, clinical outcome, nutritional status, previous antibiotic use, sample volume, and length of hospital stay. To the best of our knowledge, this is the first study on TTP from SSA.

We observed that at 24, 36, 48, and 72 hours after incubation, 70% (95% CI, 65 - 74), 76% (95% CI, 72 - 79), 89% (95% CI, 86 - 92), and 96% (95% CI, 94 - 97), respectively, of blood cultured had turned positive. This finding indicates that most blood cultures would turn positive within 48 hours of incubation. Before the introduction of automated continuous monitoring blood culture systems, traditional blood culturing methods requiring manual techniques were used. A generally acceptable minimum of 48 hours of observation time after the collection of blood samples in hospitalized children with suspected sepsis was established [23,24]. After the introduction of commercially available automated continuous monitoring blood culture systems, several studies have reported a decrease in TTP [17,[25], [26], [27]]. A large study conducted in 17 centers in the United States to determine the TTP in febrile hospitalized infants showed that at 36 hours, 96% of pathogenic blood cultures had turned positive [27]. Similar to suggestions from other studies, a maximum of 48 hours of observation after the commencement of antibiotic therapy in hospitalized children may be sufficient compared to the generally accepted minimum “48-72 hours rule” of observations [17]. This could enable clinicians to make informed decisions on antibiotic treatment within the shortest possible time.

The median TTP of 19.2 hours in our study is similar to that found in a study conducted among pediatric patients in the United States (19.8 hours) [25] but higher than in studies conducted in patients of all ages in a tertiary care university hospital in Spain (7.3 hours) [13], in children aged 0-16 years in 10 major pediatric hospitals in Switzerland (11.7 hours) [17], and in infants aged 90 days or younger across 17 pediatric centers in the United States (13.0 hours) [27]. The differences in patient populations, the actual volume of blood used to inoculate bottles, transportation times, type of sample, laboratory working hours, time to load bottles in a machine, and source of infection may explain the variation in the TTP [21].

In our study, TTP was dependent on the pathogen and independent of age, sex, temperature at presentation, clinical outcome, nutritional status, and length of hospital stay. In contrast to previous studies that have found evidence of TTP as a predictor of clinical outcomes, such as mortality and length of hospital stay [[11], [12], [13],^28^], we found no such evidence. Previous studies have reported findings similar to ours [17,29]. *S. pneumoniae* had the shortest median TTP (17.4 hours) and *Neisseria* species took the longest time to grow (45 hours)*.* Patients with *S. pneumoniae* infections had favorable clinical outcomes. Perhaps this reflects prompt and appropriate antibiotic treatment. Previous studies on TTP have shown that TTP varies by pathogen type. Although some studies reported that gram-negative bacteria have shorter TTP than gram-positive bacteria [25,30], we found that gram-positive pathogens had a shorter TTP in our study. This may reflect the distribution of pathogens and local epidemiological factors. A study by Olatunji *et al.* showed that 55% of invasive bacteria diseases in our study area were caused by gram-positive bacteria [31]. This may explain the findings of our study.

Blood cultures from children who died of *S. aureus* infection took longer to grow (GMR 1.5, [95% CI: 1.1 - 2.2]) than those who fully recovered. A previous study conducted in patients in a large Canadian health region has reported a relationship between longer TTP of *S. aureus* bacteremia and adverse clinical outcomes [11].

Our study has several strengths. We analyzed 547 blood cultures with confirmed invasive bacteria pathogens collected from children under 5 years from 11 health facilities, including two main hospitals across two regions in the Gambia. This is a large population-based study, and our results can be generalized to the rural population in the Gambia. To the best of our knowledge, this is the first study from Africa that has assessed host and pathogen factors associated with TTP in children under 5 with positive blood cultures admitted to health facilities. Our study adds to the limited data from Africa, which has one of the highest burdens of infectious diseases.

There are some important limitations to our study. First, confounding factors, such as the volume of blood collected, the time between blood collection and placement in the automated machine, and previous use of antibiotics before blood sample collection, are known to affect bacterial growth [[32], [33], [34], [35]]. To investigate the potential influence of the time lapse between blood collection and placement in the automated machine, the sensitivity analyses indicated no difference in TTP across all indicators (Supplementary Table 1). Although we explored the effect of blood volume on TTP, our analysis suggested no impact on TTP (Table 1). In addition to collecting data on self-reported antibiotic use, we also performed antibiotic activity detection testing on plasma samples from patients seen at the two hospitals. However, results were available for only 61 of 418 samples and analyses of the limited data and the self-reported antibiotic data showed no effect on TTP. The missing data on antibiotic activity testing may potentially bias the TTP. Notwithstanding, excluding these patients would not reflect real-world clinical scenarios and, thus, our data are more pragmatic [17]. Second, we excluded patients with polymicrobial culture because we could not determine the contribution of each pathogen to the TTP. Third, we excluded culture-positive cases with missing laboratory and clinical data and organisms designated as contaminants because these are not recognized as disease-causing organisms. The relatively moderate number of missing data may potentially bias the point estimates. Finally, we were unable to perform species identification for some of the coliforms because of logistical reasons and this may underestimate the prevalence of some of the pathogens.

## Conclusion

In rural Gambia, most blood cultures from pediatric patients would turn positive within 48 hours of incubation. A maximum of 48 hours of observation after the commencement of antibiotic therapy in hospitalized children may be sufficient in contrast to the generally accepted minimum of 48 hours of observation. Although TTP may be associated with the category of gram of the bacteria, TTP was not correlated with mortality or length of hospital stay in our study. Further prospective studies in patients of all ages may be required to assess the utility of TTP as a prognostic tool.

## Declarations of competing interest

The authors have no competing interests to declare.

## Appendix Supplementary materials

Image, application 1
