# Supplementary material for: Blood culture time to positivity in pediatric patients with bloodstream infection in rural Gambia
Source: IJID Reg. 2025 Feb 18;14:100606. doi: 10.1016/j.ijregi.2025.100606 (PMC11923810; doi:10.1016/j.ijregi.2025.100606)
Supplement: Supplementary file 1 [file mmc1.docx]

Supplementary table 1:

Characteristics and TTP excluding samples collected on different dates to the date of incubation

| Characteristic | N^*^ (%) | TTP, Median (IQR), h | | P value (LRT) |
| --- | --- | --- | --- | --- |
| Total No. | 504 (100) | 19.1 (16.6 – 29.8) | |  |
| *Age at blood draw, m* |  |  |  | |
| Neonate | 182 (36.1) | 19.9 (16.8 – 38.8) | |  |
| 1-11 months | 147 (29.2) | 19.0 (16.5 – 37.6) | | 0.96 |
| 12-23months | 110 (21.8) | 18.8 (16.2 – 28.3) | |  |
| 24-59months | 65 (12.9) | 18.3 (16.3 – 27.8) | |  |
| *Sex* |  |  | |  |
| Male | 272 (54.0) | 19.0 (16.3 – 26.8) | |  |
| Female | 232 (46.0) | 19.2 (16.7 – 38.6) | | 0.52 |
| *Temperature at blood draw, °C* |  |  | |  |
| <38.0 | 302 (59.9) | 19.1 (16.6 – 28.3) | |  |
| ≥38.0 | 202 (40.1) | 19.0 (16.6 – 34.6) | | 0.48 |
| *Clinical outcome* |  |  | |  |
| Alive | 445 (88.3) | 19.2 (16.4 – 29.2) | |  |
| Dead | 59 (11.7) | 19.1 (16.3 – 39.2) | | 0.42 |
| *Severe malnutrition, (Zscore<-3SD)* |  |  | |  |
| Yes | 133 (28.7) | 19.3 (16.7 – 28.9) | |  |
| No | 330 (71.3) | 18.9 (16.4 – 27.6) | | 0.61 |
| *Length of hospital stay, d* |  |  | |  |
| <3 days | 192 (39.0) | 19.1 (16.9 – 32.1) | |  |
| 3 - 6 days | 213 (43.2) | 19.0 (16.2 – 24.8) | | 0.53 |
| ≥7 days | 17 (17.8) | 19.0 (16.0 – 40.3) | |  |
| *Self-reported antibiotic use within past one week* |  |  | |  |
| No | 390 (78.8) | 19.1 (16.6 – 28.9) | |  |
| Yes | 105 (21.2) | 19.4 (16.4 – 32.8) | | 0.54 |
| ^#^*Antibiotic activity detected in sample* |  |  | |  |
| Absent | 50 (87.7) | 19.5 (16.1 - 23.5) | |  |
| Present | 7 (12.3) | 16.2 (16.1 – 21.5) | | 0.42 |
| *Type of health facility* |  |  | |  |
| Health centre | 111 (22.0) | 19.0 (16.7 – 38.9) | |  |
| Hospital | 393 (78.0) | 19.1 (16.5 – 27.2) | | 0.19 |
| *Volume of blood sample, mls* |  |  | |  |
| ≤ 1.5 | 213 (46.8) | 18.4 (16.3 – 27.2) | |  |
| > 1.5 | 242 (53.2) | 19.3 (16.7 – 27.7) | | 0.72 |
| *Pathogen category* |  |  | |  |
| Gram-positive | 203 (40.3) | 18.6 (16.2 – 23.5) | |  |
| Gram-negative | 301 (59.7) | 19.3 (16.7 – 39.6) | | 0.01 |

*Missing values Severe malnutrition = 41; Length of hospital stay = 11; antibiotic use=9, antibiotic activity detected=336; volume of blood sample=49

TTP = Time to Positivity

# Assessed only in children who presented to the hospital

LRT = Likelihood ratio test.
